# Influenza Infection During Pregnancy and Risk of Seizures in Offspring

**DOI:** 10.1001/jamanetworkopen.2024.34935

**Published:** 2024-09-23

**Authors:** Yi-Feng Lee, Yi-Hsuan Lin, Ching-Heng Lin, Ming-Chih Lin

**Affiliations:** 1 Children’s Medical Center, Taichung Veterans General Hospital, Taichung, Taiwan; 2 Department of Post-Baccalaureate Medicine, College of Medicine, National Chung Hsing University, Taichung, Taiwan; 3 School of Medicine, National Yang Ming Chiao Tung University, Taipei, Taiwan; 4 Department of Medical Research, Taichung Veterans General Hospital, Taichung, Taiwan; 5 Department of Food and Nutrition, Providence University, Taichung, Taiwan; 6 School of Medicine, College of Medicine, Chung Shan Medical University, Taichung, Taiwan

## Abstract

**Importance:**

Seizure is a common neurological problem among infants and children up to age 6 years. Prenatal exposure to maternal influenza infection has been reported to be associated with childhood seizures.

**Objective:**

To evaluate the association between maternal influenza infection and risk of childhood seizures.

**Designs, Setting, and Participants:**

This cohort study identified mother-offspring pairs from January 1, 2004, to December 31, 2013, using records in Taiwan’s Maternal and Child Health Database. Mothers who had influenza infection during pregnancy and their first offspring were identified and assigned to the influenza group. Mothers in the control group were those without influenza during pregnancy and were matched 1:4 with mothers in the influenza group by maternal age, offspring sex, and date of delivery. Offspring were followed up until December 31, 2020. Data were analyzed between March 2023 and July 2024.

**Exposure:**

Diagnosis of influenza infection during pregnancy defined using *International Classification of Diseases, Ninth Revision, Clinical Modification* codes 487.0, 487.1, and 487.8, or *International Statistical Classification of Diseases, Tenth Revision, Clinical Modification* codes J09, J10, and J11.

**Main Outcomes and Measures:**

The primary outcome was the association between maternal influenza infection during pregnancy and risk of any type of seizures during childhood, including both epilepsy and febrile seizures. Adjusted hazard ratios (AHRs) and 95% CIs were estimated using Cox proportional hazards regression models. Pregnancy-related complications were collected as covariates.

**Results:**

A total of 1 316 107 mother-offspring pairs were enrolled, of whom 75 835 mothers (predominant maternal age, 25-29 years; 39 324 male offspring [51.9%]) were assigned to the influenza group and 1 240 272 were matched and assigned to the control group (n = 303 340; predominant maternal age, 30-34 years; 157 296 male offspring [51.9%]). In the influenza group, there was a slightly higher prevalence of placenta previa or abruption compared with the control group (1.6% [1241] vs 1.4% [4350]; *P* < .001). The cumulative risk of seizures was higher among offspring whose mothers had influenza infection. After controlling for potential confounders, the AHRs were 1.09 (95% CI, 1.05-1.14) for seizures, 1.11 (95% CI, 1.06-1.17) for febrile convulsions, and 1.04 (95% CI, 0.97-1.13) for epilepsy. In the subgroup analysis, no statistically significant differences were observed between the trimesters regarding the timing of influenza infection.

**Conclusions and Relevance:**

Results of this cohort study suggest that maternal influenza infection during pregnancy was associated with an increased risk of childhood seizures, especially febrile seizures, but not epilepsy. Further studies are needed to elucidate the mechanisms underlying childhood neurological development.

## Key Points

**Question:**

Is influenza during pregnancy associated with a higher risk of seizures among offspring?

**Findings:**

In this cohort study of 1 316 107 mother-offspring pairs, maternal influenza infection during pregnancy was associated with an increased risk of febrile seizures but not epilepsy.

**Meaning:**

These findings suggest the need for future studies that elucidate the mechanisms underlying childhood neurological development.

## Teaser

This cohort study examines the association between maternal influenza infection during pregnancy and development of seizures in offspring.

## Introduction

Seizure is defined as uncontrolled, sudden electrical activity in the brain, resulting in alterations in movements, behavior, feelings, and consciousness status.^1,2^ Febrile seizure, also termed *febrile convulsion*, is the most common neurologic problem among infants and children up to age 6 years. It is characterized by a convulsion associated with an elevated body temperature without other provoking factors.^3,4^ Febrile seizures occur in 2% to 5% of all children in Western countries.^5,6^ The incidence rate varies in East Asia from 1.0% in China, 2.4% to 4.2% in Taiwan, 8.2% in Japan, to 14.0% in Guam.^7,8,9,10^

The developmental origins of health and disease hypothesis posits that periconceptual, prenatal, and early extrauterine environmental stressors have implications for the lifetime health of the offspring.^11^ Maternal bacterial and viral infections during pregnancy have preceded certain neurological sequelae in children, including preterm birth, neurodevelopmental or behavioral disorders, childhood epilepsy, and other systemic disease.^12,13,14^ Influenza infection during pregnancy has been associated with adverse birth outcomes.^15,16^ Prenatal exposure to maternal influenza infection has been reported as a factor in childhood seizures in Western countries.^17^ However, to date, no population-based study on this issue has been conducted in East Asian countries. The aim of this study was to explore the association between maternal influenza infection and childhood seizures from a population-based perspective.

## Methods

### Data Sources and Study Design

This was a nationwide cohort study. The Taichung Veterans General Hospital Institutional Review Board approved this study and waived the informed consent requirement because publicly available, deidentified data were used. We followed the Strengthening the Reporting of Observational Studies in Epidemiology (STROBE) reporting guideline.

The primary source of data was Taiwan’s Maternal and Child Health Database (MCHD), a comprehensive repository managed by the Health and Welfare Data Science Center of Taiwan’s Ministry of Health. The MCHD links several vital datasets, including the Taiwan Birth Registration Database, Birth Certificate Application, National Register of Death, and the National Health Insurance Research Database (NHIRD). By combining these datasets, MCHD can provide integrated medical claims data pertaining to both the offspring and their parents. The NHIRD encompasses extensive information from Taiwan’s National Health Insurance program, covering over 99% of the country’s population of approximately 23 million individuals. The NHIRD contains each patient’s demographic characteristics, residence, registration (type of occupation), health examinations, diagnoses, medical procedures, surgeries, medication prescriptions, and medical expenditures. The main dataset used in the present analysis was obtained from the NHIRD and comprised inpatient expenditures by admission and ambulatory care expenditures by visit.

All medical records were derived from outpatient services, hospitalization documents, and emergency department visits.^18^ Eligible mother-offspring pairs between January 1, 2004, and December 31, 2013, were linked by birth notification file. Only mothers and their first offspring were selected in this period to avoid a cluster effect from the same families. The offspring were followed up until December 31, 2020. We excluded mothers with a history of epilepsy to minimize its implications for the study results. Each gestational age was ascertained by an obstetrician and was included in the Taiwan Birth Registration Database. In the past, several studies with similar designs used population databases to link mothers and offspring.^19^ The NHIRD has been broadly used in research. Moreover, the history, validation, database linkage, data access, application, ethics, confidentiality, strengths, and weaknesses of the NHIRD have been well established, as described in a review article.^20^

### Maternal Influenza Infection

Primiparous mothers who had an outpatient visit, an emergency department visit, or hospitalization with the main diagnosis of influenza during the study period were recruited. These mothers were assigned to the influenza group. Mothers in the control group were those without influenza during pregnancy and matched 1:4 with the mothers in the influenza group by maternal age, offspring sex, and date of delivery. The diagnosis of influenza infection during pregnancy was defined using *International Classification of Diseases, Ninth Revision, Clinical Modification* (*ICD-9-CM*) codes 487.0, 487.1, and 487.8 or *International Statistical Classification of Diseases, Tenth Revision, Clinical Modification* (*ICD-10-CM*) codes J09, J10, and J11. To identify the potential role of maternal infection timing in the subsequent association of childhood seizures, the mothers were further stratified into subgroups according to contracting influenza in different trimesters.

### Outcome 

The medical records of offspring were followed up until December 31, 2020, to ensure an observation period of at least 7 years and up to 16 years. The primary outcome of the study was a diagnosis of any type of seizures during childhood, including epilepsy (*ICD-9-CM* codes 345.00 to 345.91 or *ICD-10-CM* codes G40 and G40.9) and febrile seizures (*ICD-9-CM* codes 780.31 and 780.39 or *ICD-10-CM* code R56). Offspring who had seizures, including epilepsy and febrile seizures, would be counted in the category called *all seizures*. If the offspring initially experienced febrile seizures and were subsequently diagnosed with epilepsy, they would be included in both categories. Offspring with an emergency department visit, hospitalization, or 3 outpatient visits with a main diagnosis of seizure were regarded as having the disease.

### Covariates 

Pregnancy-related complications were collected as potential confounding factors. These complications included gestational hypertension (*ICD-9-CM* codes 648.0 and 648.8 or *ICD-10-CM* code O24), gestational diabetes (*ICD-9-CM* code 642.3 or *ICD-10-CM* codes O13 and O16), preeclampsia or eclampsia (*ICD-9-CM* codes 624.4, 624.5, and 624.6 or *ICD-10-CM* codes O11, O14, and O15), placenta previa or abruption (*ICD-9-CM* code 641 or *ICD-10-CM* codes O44 and O45), and anemia (*ICD-9-CM* code 648.2 or *ICD-10-CM* codes O99.019, O99.011, O99.012, O99.013, O99.020, and O99.030).

### Statistical Analysis

Demographic data were expressed as percentages and compared using the χ^2^ test. Kaplan-Meier estimates with log-rank tests and Cox proportional hazards regression models, which were adjusted for potential confounding factors, including maternal age, family income, urbanization, pregnancy-related complication, mode of delivery, offspring sex, multiple birth, birth weight, and gestational age, were applied to compare the cumulative hazards among groups. Sensitivity analysis of the adjusted hazard ratio (AHR) for seizures was conducted by multivariable Cox proportional hazards regression models. All data were analyzed between March 2023 and July 2024 using SAS, version 9.4 (SAS Institute Inc). A 2-sided *P* < .05 was considered statistically significant.

## Results

### Basic Demographics 

**Figure 1.**
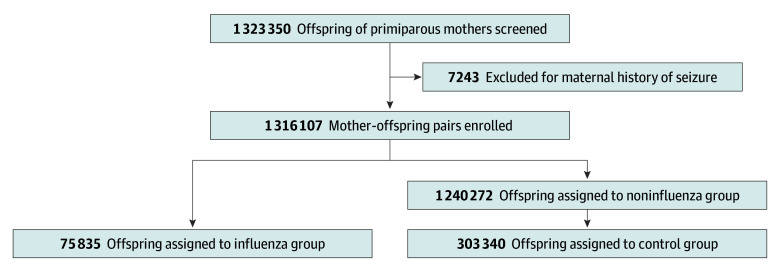
Composition of the Study Cohort

Between 2004 and 2013, 1 323 350 offspring of primiparous mothers in Taiwan were screened using the NHIRD. After exclusion of 7243 offspring whose mothers had a history of seizures, 1 316 107 mother-offspring pairs were enrolled. A total of 75 835 offspring (36 511 females [48.1%], 39 324 males [51.9%]; predominant maternal age, 25-29 years) whose mothers were diagnosed with influenza infection during pregnancy were assigned to the influenza group, while 1 240 272 offspring whose mothers did not have influenza during pregnancy were assigned to the noninfluenza group. The noninfluenza group was then matched with the influenza group, which yielded the control group (n = 303 340), of whom 146 044 were female (48.1%) and 157 296 were male (51.9%) offspring (Figure 1). The basic demographic characteristics of these mother-offspring pairs are summarized in Table 1. Among the pregnancy-related complications, offspring in the influenza group had a slightly higher prevalence of placenta previa or abruption compared with the control group (1.6% [1241] vs 1.4% [4350]; *P* < .001).

**Table 1.** Characteristics of Mother-Offspring Pairs

Characteristic	Mother-Offspring Pairs, No. (%)		Total No.	*P* value
	Control group (n = 303 340)	Influenza group (n = 75 835)		
Maternal age, y				
<25	65 680 (21.7)	16 420 (21.7)	82 100	>.99
25-29	113 772 (37.5)	28 443 (37.5)	142 215	
30-34	90 408 (29.8)	22 602 (29.8)	113 010	
≥35	33 480 (11.0)	8370 (11.0)	41 850	
Family income, $				
≤18 780	99 086 (32.7)	25 177 (33.2)	124 263	<.001
18 781-27 600	104 690 (34.5)	29 644 (39.1)	134 334	
27 601-42 000	63 608 (21.0)	14 310 (18.9)	77 918	
>42 000	35 956 (11.9)	6704 (8.8)	42 660	
Urbanization				
Urban	180 879 (59.6)	44 078 (58.1)	224 957	<.001
Suburban	40 102 (13.2)	11 179 (14.7)	51 281	
Rural	82 359 (27.2)	20 578 (27.1)	102 937	
Pregnancy-related complication				
Gestational hypertension	299 (0.1)	74 (0.1)	373	.93
Gestational diabetes	3059 (1.0)	752 (1.0)	3811	.67
Preeclampsia or eclampsia	684 (0.2)	183 (0.2)	867	.41
Placenta previa or abruption	4350 (1.4)	1241 (1.6)	5591	<.001
Anemia	1695 (0.6)	451 (0.6)	2146	.23
Mode of delivery				
Vaginal	200 881 (66.2)	47 995 (63.3)	248 876	<.001
Cesarean	102 459 (33.8)	27 840 (36.7)	130 299	
Offspring sex				
Female	146 044 (48.1)	36 511 (48.1)	182 555	>.99
Male	157 296 (51.9)	39 324 (51.9)	196 620	
No. delivered				
Singleton	298 455 (98.4)	74 830 (98.7)	373 285	<.001
Multiple	4885 (1.6)	1005 (1.3)	5890	
Birth weight, g				
≥2500	281 535 (92.8)	70 638 (93.1)	352 173	<.001
2000-2499	16 558 (5.5)	4102 (5.4)	20 660	
<2000	5247 (1.7)	1095 (1.4)	6342	
Gestational age, wk				
≥37	279 469 (92.1)	69 954 (92.2)	349 423	<.001
32-36	21 654 (7.1)	5460 (7.2)	27 114	
26-31	1876 (0.6)	354 (0.5)	2230	
<26	341 (0.1)	67 (0.1)	408	

### Risk of Seizure in Offspring in the Influenza Group 

**Figure 2.**
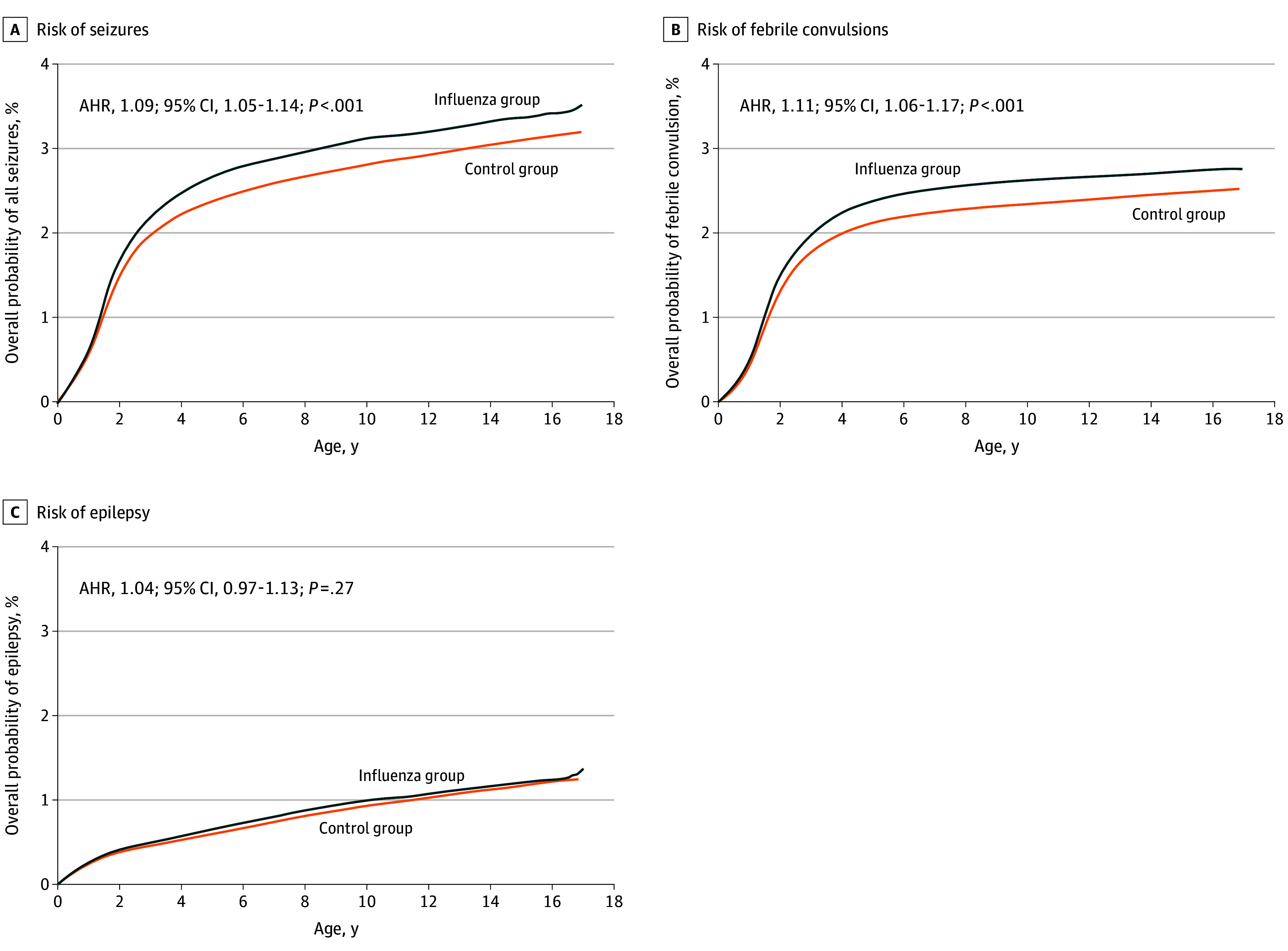
Cumulative Risks of Seizures, Febrile Convulsions, and Epilepsy in the Influenza vs Control Group The risks of seizures and febrile convulsions were higher in offspring in the influenza group, while the risk of epilepsy did not follow a similar pattern.

Among the 75 835 offspring whose mothers had influenza infection during pregnancy, 2456 (3.2%) developed seizures during their follow-up period. The cumulative risk of seizures in the influenza group was higher than in the control group (Figure 2A). After stratification by seizure types, the cumulative risk of febrile convulsions was significantly higher in offspring of mothers with influenza during pregnancy (Figure 2B). However, the risk of epilepsy did not follow a similar pattern (Figure 2C). After controlling for potential confounders using Cox proportional hazards regression models, the AHRs were 1.09 (95% CI, 1.05-1.14) for seizures, 1.11 (95% CI, 1.06-1.17) for febrile convulsions, and 1.04 (95% CI, 0.97-1.13) for epilepsy in offspring in the influenza group (Figure 2; Table 2). Results of the Cox proportional hazards regression models revealed that for all seizures the following covariates were significant: maternal age (eg, age 25-29 years vs <25 years AHR, 0.90; 95% CI 0.86-0.94; *P* < .001), gestational hypertension (AHR, 1.78; 95% CI 1.18-2.69; *P* < .001), mode of delivery (cesarean delivery AHR, 1.09; 95% CI 1.05-1.14; *P* < .001), male offspring sex (AHR, 1.28, 95% CI 1.23-1.32; *P* < .001), birth weight (eg, 2000-2499 g vs ≥2500 g AHR, 1.27; 95% CI 1.17-1.38; *P* < .001), and gestational age (eg, 32-36 weeks vs ≥37 weeks AHR, 1.23; 95% CI 1.14-1.33; *P* < .001). Multiple births was not significant for all seizures (AHR, 0.87; 95% CI 0.76-1.00; *P* = .06).

**Table 2.** Risk and Hazard Ratio of Seizures for Offspring in the Influenza Group

Outcome	No. (%)		*P* value	Risk of seizure for influenza group, AHR (95% CI)^a^	*P* value
	Control group (n = 303 340)	Influenza group (n = 75 835)			
Epilepsy	3216 (1.1)	839 (1.1)	.26	1.04 (0.97-1.13)	.27
Febrile convulsion	7302 (2.4)	2024 (2.7)	<.001	1.11 (1.06-1.17)	<.001
All seizures	8989 (3.0)	2456 (3.2)	<.001	1.09 (1.05-1.14)	<.001

Abbreviation: AHR, adjusted hazard ratio.

^a^ AHR calculated by multivariable Cox proportional hazards regression models.

### Trimester Analysis

A slight increase in the risk of seizures, febrile convulsions, and epilepsy during the third trimester was observed. However, the increases were not significant (Table 3).

**Table 3.** Multivariable Cox Proportional Hazards Regression Models of Seizure Stratified by Trimesters

Trimester	Epilepsy			Febrile convulsion			All seizures		
	Case, No. (%)	AHR (95% CI)	*P* value	Cases, No. (%)	AHR (95% CI)	*P* value	Cases, No. (%)	AHR (95% CI)	*P* value
1 (<13 wk)	342 (1.1)	1 [Reference]	NA	829 (2.7)	1 [Reference]	NA	1005 (3.2)	1 [Reference]	NA
2 (13-27 wk)	336 (1.1)	1.00 (0.86-1.16)	.98	832 (2.7)	1.01 (0.92-1.12)	.78	1003 (3.3)	1.01 (0.93-1.10)	.82
3 (≥28 wk)	161 (1.2)	1.12 (0.93-1.35)	.25	363 (2.7)	1.02 (0.90-1.15)	.77	448 (3.3)	1.04 (0.93-1.16)	.48

Abbreviation: AHR, adjusted hazard ratio.

## Discussion

The results of this cohort study revealed an increased risk of childhood seizures, particularly febrile seizures but not epilepsy, in offspring born to mothers who were diagnosed with influenza infection during pregnancy. Exposure to infection in utero may have long-term consequences for the offspring, especially with respect to development of neuropsychiatric disorders. A Swedish cohort study demonstrated that maternal exposure to infection while hospitalized increased the risk of autism and depression in offspring during the follow-up period.^21^ However, there are conflicting findings in the literature on maternal influenza infection.^22^ Recently, an international study that included Norway, Australia, and Canada found that prenatal exposure to influenza infection increased the risk of childhood seizures (HR, 1.17; 95% CI, 1.07-1.28). The risk of febrile seizure also increased (HR, 1.20; 95% CI, 1.07-1.34). However, the risk of epilepsy did not increase (HR, 1.07; 95% CI, 0.81-1.41).^17^

These primary findings align closely with the results of the present study. The trimester analysis also showed that the risk estimates for seizures were notably higher after influenza infection during the second and third trimesters compared with the first trimester.^17^ Although we observed slight increases in the risk of seizures, febrile convulsions, and epilepsy during the third trimester, there were no associations. A meta-analysis found that no conclusion could be reached regarding the association between maternal influenza infection and childhood neuropsychiatric outcomes due to the limited quantity and quality of available research.^23^ We also conducted a sensitivity analysis of the HR for seizures using multivariable Cox proportional hazards regression models. After adjustment for all maternal factors, there was no significant difference in the AHRs. Regardless of whether the models were adjusted for maternal age, family income, urbanization, or pregnancy-related complications, the results remained consistent, demonstrating the robustness of the study findings. The relevant data were summarized in eTable in Supplement 1.

As febrile seizures typically occur between 6 months and 5 years, the chance of loss to follow-up should be low. The reported cumulative incidence of febrile convulsion ranged from 2.4% before age 3 years,^8^ 3.2% between ages 6 months and 6 years, to 4.2% between ages 3 and 6 years.^9,24^

During pregnancy, it is broadly believed that a state of generalized immunosuppression develops, which makes the mother more susceptible to certain infectious diseases.^25^ These pathogens may have unfavorable fetal and neonatal consequences, due to intrauterine transmission via the placenta. These outcomes include stillbirth, neonatal death, congenital anomalies, intraventricular hemorrhage, respiratory distress syndrome, necrotizing enterocolitis, cerebral palsy, intellectual disability, preterm birth, and fetal growth restriction.^26^ Moreover, evidence indicates that the maternal immune system must undergo complex modifications to establish and maintain tolerance toward the fetus, while simultaneously providing protection against certain pathogens.^27^ Maternal immune activation and placental inflammation may be associated with inadequate placental perfusion and intrauterine hypoxia, which have been linked to adverse fetal neurodevelopmental outcomes.^28,29,30^ Moreover, there is growing evidence that the imbalanced maternal immune status attributed to infection rather than the pathogens themselves may play a crucial role in fetal neurological developmental issues.^31,32^ Cytokine storm induced by maternal influenza infection has also been proposed as a possible pathogenetic mechanism.^33^

An animal study demonstrated that maternal respiratory influenza virus infection could adversely affect offspring behavior.^34^ Affected mice displayed decreased exploratory behavior compared with controls, and these changes were attributed to the maternal immune response rather than the viral infection itself. General inflammatory changes in vessels, vascular dysfunction, with increased proinflammatory cells and cytokines were found in pregnant mice with influenza A virus infection. This phenomenon can lead to poor placental maturation, restricted fetal growth, and molecular evidence of fetal brain hypoxia.^35^ Gray matter hypoxic-ischemic neuronal damage could be induced through cord occlusion in fetal sheep, and susceptibility to this damage increased with advancing gestation.^36^ Repetitive umbilical cord occlusions could have detrimental consequences for neuronal electrocortical brain activity.^37^ Hypoxia-affected gray matter is more electrosensitive and could precede a seizure attack. These findings might explain, at least in part, why offspring in the influenza group had a greater risk for seizures. Additionally, suppression of fetal thymic genes has been found in mice with maternal influenza infection.^38^ It potentially renders offspring more susceptible to infections, and it may partially explain the reason that maternal influenza infection is associated with increased risk of febrile convulsions.

### Limitations

This study has several limitations. First, due to patient privacy protocols, the validity of coding in the NHIRD could not be examined. Thus, misclassifications of exposure and outcome may have existed. In this study, maternal influenza infection during pregnancy was defined using *ICD* codes, which were recorded during outpatient visits, emergency department visits, or hospitalizations. The diagnosis of influenza is typically established from a positive influenza test result, flu-like symptoms, and/or contact history. Although medical access is easy and convenient within Taiwan’s health care system, some pregnant individuals may avoid seeing a physician due to mild symptoms or a fear of taking medication. Misclassification of influenza exposure may have occurred, but its implications for the findings were likely minimal, as the incidence of influenza infection during pregnancy in this study mirrored rates in prior population-based studies. For instance, in a large population-based study in 2009, 368 of 107 889 pregnant women (0.3%) were diagnosed with influenza during a period of seasonal influenza virus predominance.^39^ Additionally, the incidence rates were 88.7 per 10 000 pregnant individual–months during the 2017 season and 69.6 per 10 000 pregnant individual–months during the 2018 season in a population-based study conducted in 3 middle-income countries by the US Centers for Disease Control and Prevention.^40^ The outcome misclassification should be minimal because of the convenient medical access in Taiwan. Parents tend to take their children to an emergency department when seizures occur. Even if there were certain cases of seizures that were not coded correctly by physicians, the misclassification should be nondifferential.

Second, the records of influenza vaccination and antiviral medication throughout the pregnancy period were not available for the study because of the different public health policies that were implemented annually in Taiwan. When vaccines or medications were paid for out of pocket, they may not have been documented in the insurance database, leading to fluctuating and unreliable numbers in the records.

Third, Taiwan is an isolated island with a National Health Insurance program that covers nearly all of its population. Thus, the lost-to-follow-up rate should be minimal. However, the exact data on offspring lost to follow-up due to death or emigration were not available for this study. This information might decrease the statistical power for this study.

Fourth, we considered only maternal covariates and covariates and did not analyze the comorbidities and health conditions specific to the offspring. After all, the study aimed to explore the developmental origins of health and disease hypothesis^11^ that prenatal maternal exposure to infection is associated with neurological issues in affected offspring. Furthermore, the comorbidities and health conditions of the offspring might play a role as mediators to outcomes.

Fifth, we did not include information on the severity of influenza, such as cases requiring hospitalization or ventilators. However, influenza infection needing hospitalization or ventilators is relatively rare. We will consider addressing this issue in future research.

Sixth, mothers with epilepsy have been shown to confer an increased risk of childhood seizures^41^ and were believed to be more likely to seek health care; they may also be overrepresented in this study’s influenza group. Therefore, we excluded mothers with a history of epilepsy. However, whether these mothers are more likely to seek health care remains unknown and is an interesting question. Further studies should be conducted to explore this issue.

## Conclusions

The findings of this study suggest that maternal influenza infection during pregnancy might increase the risk of childhood seizures, especially febrile seizures, but not epilepsy. Further studies are needed to elucidate the mechanisms underlying childhood neurological development.

## Notes

Supplement 1. **eTable.** Sensitivity Analysis Using Multivariable Cox Proportional Hazards Regression Models

Supplement 2.
Data Sharing Statement
