# Supplementary material for: Influenza Infection During Pregnancy and Risk of Seizures in Offspring
Source: JAMA Netw Open. 2024 Sep 23;7(9):e2434935. doi: 10.1001/jamanetworkopen.2024.34935 (PMC11420688; doi:10.1001/jamanetworkopen.2024.34935)
Supplement: Supplement 1. — eTable. Sensitivity Analysis Using Multivariable Cox Proportional Hazards Regression Models [file jamanetwopen-e2434935-s001.pdf]

Supplemental Online Content

Lee Y-F, Lin Y-H, Lin C-H, Lin M-C. Influenza infection during pregnancy and risk of seizures in offspring. *JAMA Netw Open*. 2024;7(9):e2434935. doi:10.1001/jamanetworkopen.2024.34935

eTable. Sensitivity analysis using multivariable Cox proportional hazards regression models

This supplemental material has been provided by the authors to give readers additional information about their work.

© 2024 Lee Y-F et al. *JAMA Network Open*

eTable. Sensitivity analysis using multivariable Cox proportional hazards regression models

Characteristic	Epilepsy				Febrile convulsion				All seizures			
	HR	95%CI		p-value	HR	95%CI		p-value	HR	95%CI		p-value
**Influenza group**	1.04	0.97	1.13	0.27	1.11	1.06	1.17	<.001	1.09	1.05	1.14	<.001

Characteristic	Epilepsy				Febrile convulsion				All seizures			
	HR	95%CI		p-value	HR	95%CI		p-value	HR	95%CI		p-value
**Influenza group**	1.04	0.97	1.13	0.27	1.11	1.06	1.17	<.001	1.09	1.05	1.14	<.001
**Maternal age**												
<25	1.00				1.00				1.00			
25-29	0.87	0.81	0.95	0.001	0.88	0.84	0.93	<.001	0.89	0.85	0.93	<.001
30-34	0.91	0.83	0.99	0.026	0.83	0.79	0.88	<.001	0.86	0.82	0.91	<.001
≥35	1.00	0.90	1.12	0.99	0.81	0.75	0.88	<.001	0.88	0.82	0.94	<.001

Characteristic	Epilepsy				Febrile convulsion				All seizures			
	HR	95%CI		p-value	HR	95%CI		p-value	HR	95%CI		p-value
**Influenza group**	1.04	0.96	1.12	0.32	1.11	1.05	1.16	<.001	1.09	1.04	1.14	<.001
**Maternal age**												
<25	1.00				1.00				1.00			

© 2024 Lee Y-F et al. *JAMA Network Open*

25-29	0.89	0.81	0.96	0.004	0.90	0.85	0.94	<.001	0.90	0.86	0.95	<.001
30-34	0.93	0.85	1.02	0.12	0.86	0.81	0.91	<.001	0.89	0.84	0.94	<.001
≥35	1.03	0.92	1.15	0.62	0.84	0.78	0.91	<.001	0.91	0.85	0.97	0.006
**Family income**												
$≤18780	1.00				1.00				1.00			
$18781-27600	0.94	0.87	1.01	0.09	0.98	0.94	1.03	0.47	0.98	0.94	1.02	0.37
$27601-42000	0.96	0.88	1.05	0.42	0.98	0.92	1.04	0.42	0.98	0.93	1.03	0.34
$>42000	0.85	0.76	0.96	0.008	0.87	0.81	0.94	0.001	0.86	0.81	0.93	<.001
**Urbanization**												
Urban	1.00				1.00				1.00			
Suburban	1.07	0.98	1.18	0.13	1.05	0.99	1.12	0.11	1.05	0.99	1.10	0.12
Rural	1.03	0.96	1.11	0.40	1.04	0.99	1.09	0.09	1.05	1.00	1.09	0.04
**Pregnancy-related complication**												
Gestational hypertension	1.90	0.95	3.81	0.07	2.26	1.45	3.50	<.001	2.06	1.37	3.11	<.001
Gestational diabetes mellitus	1.19	0.89	1.59	0.24	0.97	0.79	1.20	0.79	1.02	0.85	1.23	0.82
Pre-eclampsia or eclampsia	1.23	0.70	2.17	0.48	1.02	0.67	1.55	0.95	1.12	0.78	1.61	0.53
Placenta previa or abruption	1.31	1.04	1.64	0.020	1.08	0.92	1.27	0.35	1.14	0.99	1.32	0.07
Anemia	1.58	1.14	2.20	0.006	1.09	0.84	1.42	0.50	1.17	0.93	1.47	0.17

© 2024 Lee Y-F et al. *JAMA Network Open*
