# Supplementary material for: Influenza Infection During Pregnancy and Risk of Seizures in Offspring
Source: JAMA Netw Open. 2024 Sep 23;7(9):e2434935. doi: 10.1001/jamanetworkopen.2024.34935 (PMC11420688; doi:10.1001/jamanetworkopen.2024.34935)
Supplement: Supplement 2. — Data Sharing Statement [file jamanetwopen-e2434935-s002.pdf]

Data Sharing Statement

Lee. Influenza Infection During Pregnancy and Risk of Seizures in Offspring. *JAMA Netw Open*. Published September 23, 2024. doi:10.1001/jamanetworkopen.2024.34935

Data
Data available: No

Additional Information
Explanation for why data not available: To protect patients' identity and validate the reliability of the databases, investigators are required to perform onsite analysis at HWDC via remote connection to MOHW servers. Requests to access these datasets should be directed to Dr. Ching-Heng Lin, epid@vghtc.gov.tw.
